# Protocatechuic acid from chicory is bioavailable and undergoes partial glucuronidation and sulfation in healthy humans

**DOI:** 10.1002/fsn3.1168

**Published:** 2019-08-14

**Authors:** Jiakun Zheng, Haiyan Xiong, Qing Li, Luanying He, Hui Weng, Wenhua Ling, Dongliang Wang

**Affiliations:** ^1^ Department of Nutrition, School of Public Health Sun Yat‐sen University (Northern Campus) Guangzhou China; ^2^ Guangdong Provincial Key Laboratory for Food Nutrition and Health Guangzhou China; ^3^ Guangdong Province Engineering Laboratory for Nutrition Translation Guangzhou China

**Keywords:** bioavailability, chicory, humans, metabolism, protocatechuic acid

## Abstract

Protocatechuic acid exerts multiple health‐promoting effects such as anticancer, anti‐atherosclerosis, and neuroprotection in animal models. While protocatechuic acid produced in the lower gastrointestinal tract by microbial catabolism of several flavonoids is bioavailable, the pharmacokinetics of protocatechuic acid has not been evaluated so far in humans following its oral consumption. In this open‐label and single‐dose pharmacokinetic trial, 16 healthy adults followed a low‐phytochemical diet for three days. Next, after overnight fasting, participants consumed 150 g of chicory containing 248 μmol of protocatechuic acid. Blood, urine, and fecal samples were collected before and up to 24 hr after chicory consumption. Protocatechuic acid in the free and glucuronide/sulfate‐conjugated forms was almost undetectable in serum, urine, and fecal samples before chicory consumption. Chicory consumption increased the levels of protocatechuic acid and its glucuronide/sulfate conjugates in biological samples. The maximum serum concentrations of protocatechuic acid in the free‐, glucuronide‐, and sulfate‐conjugated forms were 3,273, 519, and 340 nmol/L, respectively. The recovery of total protocatechuic acid in blood circulation, urine, and feces was 23.79%, 12.17%, and 12.79% of the ingested dose, respectively. Moreover, glucuronide and sulfate conjugates of protocatechuic acid made up 34.79%, 60.15%, and 72.70% of its total recovery in blood circulation, urine, and feces, respectively. Collectively, protocatechuic acid from chicory is bioavailable and undergoes partial glucuronidation and sulfation in human adults, and its regular consumption may exert health‐promoting effects.

## 1 INTRODUCTION

Polyphenols are secondary metabolites of plants and are generally categorized as phenolic acids, flavonoids, stilbenes, and lignans (Williamson, 2017). Their compounds are widely distributed in the plant foods such as fruits, vegetables, cereals, coffee, and legumes. Regular consumption of polyphenols has been associated with a reduced risk of a number of chronic diseases including cancer and atherosclerotic diseases (Arts & Hollman, 2005; Fraga, Croft, Kennedy, & Tomas‐Barberan, 2019; Gonzalez et al., 2018). Experimental animal and in vitro mechanistic work have further unraveled that polyphenols modulate a wide range of cellular processes and thus underpin their significant health benefits in humans (Garcia‐Conesa, 2017; Gonzalez et al., 2018; Manach, Williamson, Morand, Scalbert, & Remesy, 2005).

Protocatechuic acid (3,4‐dihydroxybenzoic acid), a phenolic acid compound, is widely present in human diet, such as bran and grain brown rice, olive oil, plums, gooseberries, white grapes, star anise, chicory (*Cichorium intybus* *L. var.* *foliosum*, Belgian endive), onion, and almond (Juurlink, Azouz, Aldalati, AlTinawi, & Ganguly, 2014). However, its limited content in plant foods, in comparison with other polyphenols, has resulted in limited interest by nutritionists (Juurlink et al., 2014; Masella et al., 2012). Notably, several independent groups have recently reported that protocatechuic acid concentrations in vivo could be much higher than the simple quantity ingested because it is a major colonic microbial metabolite of several flavonoids (e.g., anthocyanins, procyanidins, and quercetin) (Masella et al., 2012; McKay, Chen, Zampariello, & Blumberg, 2015; Santangelo, Silvestrini, & Mancuso, 2019; Serra et al., 2012; Urpi‐Sarda et al., 2009; Vitaglione et al., 2007). Dietary origin and production from the colonic microbial catabolism of certain flavonoids are thus reinforcing the nutritional value of protocatechuic acid in human diet.

Extensive preclinical studies have shown that protocatechuic acid exerts multiple biological properties, such as anticancer, anti‐atherosclerosis, antidiabetes, and neuroprotection (Masella et al., 2012). Interestingly, the benefits of several flavonoids on human/animal physiologies have been shown to be partially ascribed to their metabolite protocatechuic acid (Kawabata, Yoshioka, & Terao, 2019; Liu, Wang, Lin, Ling, & Wang, 2016; Wang, Wei, Yan, Jin, & Ling, 2010; Wang, Xia, et al., 2012; Wang, Zou, Yang, Yan, & Ling, 2011). For example, we previously demonstrated that protocatechuic acid mediates the anti‐atherosclerotic effect of its precursor cyanidin‐3‐glucose effect in apolipoprotein E‐deficient (ApoE^‐/‐^) mice (Wang, Xia, et al., 2012; Wang, Zou, et al., 2011). Therefore, it is worthy to test whether the biological properties of protocatechuic acid can be extrapolated from animals to humans.

Understanding the pharmacokinetics of protocatechuic acid is a prerequisite to explain its health benefits on animals/humans, which guides the study of its bioactive compounds, identifying targets of its activity, timing of consumption, and dosing strategies to maximize its health benefit potential. Our previous studies showed that the maximal level of protocatechuic acid in plasma was approximately 4.3 µM in ApoE^‐/‐^ mice receiving a single oral gavage with 25 mg/kg of protocatechuic acid (Wang, Zou, et al., 2011). Chen et al., (2012) evaluated the pharmacokinetics of protocatechuic acid in CF‐1 mice after oral administration of 50 mg/kg protocatechuic acid and found that protocatechuic acid reached a peak plasma level of 73.6 µM. Interestingly, BALB/cA mice fed a standard diet supplemented with 2% of protocatechuic acid for 12 weeks showed an increased deposit in the blood circulation and certain tissues such as brain, heart, and kidney (Lin, Tsai, Huang, & Yin, 2011). In humans, protocatechuic acid produced in the lower gastrointestinal tract by microbial catabolism of several flavonoids that are administrated by oral consumption of ^13^C‐tracer cyanidin‐3‐glucose (Czank et al., 2013), blood orange/cranberry juice rich in anthocyanins (McKay et al., 2015; Vitaglione et al., 2007), or other plant foods/beverages (Stevens & Maier, 2016) is highly bioavailable. However, to our knowledge, the pharmacokinetics of protocatechuic acid has not been evaluated so far in humans following its oral consumption.

In the current study, we evaluated the absorption, metabolism, and excretion of protocatechuic acid in healthy adults after a single oral consumption of chicory. We selected chicory for the pharmacokinetic trial of protocatechuic acid for three reasons. Firstly, according to the Phenol‐Explorer database, perhaps the most comprehensive database on polyphenol contents, chicory ranks second only to star anise in the commonly consumed plant‐derived foods/beverages in humans in terms of protocatechuic acid content. Secondly, our preclinical studies have shown that chicory is able to recapitulate the anti‐atherosclerotic effect of its constitute protocatechuic acid (Liu et al., 2016). Last but not least, chicory is widely used as a traditional dish in the Mediterranean diet (Lin et al., 2015). We anticipate that future trials of the putative benefits of protocatechuic acid on the prevention and treatment of chronic diseases in humans can be feasibly carried out by dietary supplementation with chicory.

## 2 METHODS AND MATERIALS

### 2.1 Materials

Protocatechuic, gallic, caffeic, 5‐caffeoylquinic, and chicoric acid were purchased from Chengdu Must Bio‐Technology Co., Ltd. Caftaric acid was from Extrasynthese (Genay Cedex, France). Solutions of standard phenolic acids were prepared in 50% methanol–water (volume/volume). ‐glucuronidase (EC 3.2.1.31, type IX A from *E. coli*), sulfatase (type H‐1 from *helix pomatia*, containing ‐glucuronidase), d‐saccharic acid 1,4‐lactone (a glucuronidase inhibitor), mycophenolic acid, and brain–heart infusion were purchased from Sigma. Chicory was from Hebei Vilof Agritech Co., Ltd, which were transported in a light‐proof foam box with a cold supply chain and consumed by the subjects in 24–36 hr after harvest.

### 2.2 Subjects

We recruited healthy adult men (*n* = 8) and women (*n* = 8) participants by word of mouth among the physical examinees at the Physical Examination Center of Guangdong Second People's Hospital. The participants were nonsmokers and not taking any medication or nutritional supplements in the last three months. All participants had regular daily bowel movements. None of the women participants were pregnant. They were healthy as judged by a medical questionnaire, with normal blood values for blood pressure, aspartate aminotransferase, and alanine aminotransferase. All participants were informed about the purpose of the study and gave written informed consent before their inclusion in the trial. This trial was approved by the Ethics Committee of the School of Public Health at Sun Yat‐sen University [2017 No. 009] and registered at ClinicalTrials.gov as ChiCTR1800014393. This trial was conducted in accordance with the Declaration of Helsinki of 1975 as revised in 2008.

### 2.3 Study design and sample collection

**Figure 1:**
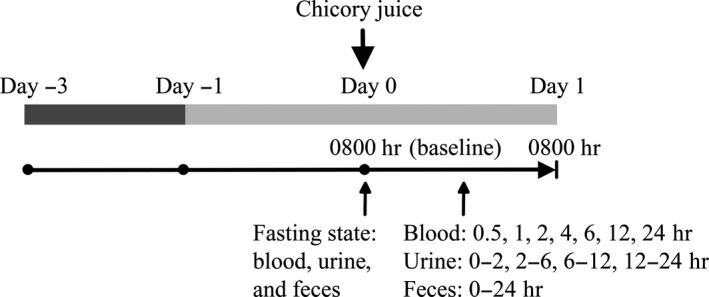
Study design. Summary of the timing of ingestion of chicory juice (down arrow) and dietary control (low‐polyphenol diet shown by dark gray; diet without fruits, vegetables, and plant‐based juices/beverages shown by light gray), and the collection of blood, urine, and feces samples (up arrows)

A single‐dose pharmacokinetic trial was conducted to evaluate the acute (24 hr) bioavailability of protocatechuic acid from chicory juice. The flowchart of participants in this pharmacokinetic trial is summarized in Figure 1. Qualified participants were asked to consume foods low in phytochemicals for three days prior to the chicory juice intervention. The restricted foods rich in anthocyanins, phenolic acids, or sesquiterpene lactones are listed in Table S1. Participants were also asked to consume a diet without vegetables, fruits, and plant‐based beverages for the day prior to the intervention and the intervention day. The purpose of these dietary restrictions was to reduce any residual dietary phenolic compounds from the body (these compounds are typically cleared from blood, urine, and feces within 48 hr of consumption) and to ensure phenolic compounds present in the blood, urine, and feces samples collected during the intervention were derived from the chicory juice alone. Prior to administering the chicory juice, overnight fasting blood, urine, and feces (first‐morning void) were collected at time 0 hr as the baseline. A single dose of chicory juice (150 g of chicory) was then orally ingested by each of the participants under close observation by the researcher in 5 min. No other foods or beverages were provided at this time. The chicory juice was prepared together approximately 10 min prior to this pharmacokinetic trial. One portion of chicory juice was kept to quantify the content of protocatechuic acid. Following administration of the chicory juice, blood samples were collected at 0.5, 1, 2, 4, 6, 12, and 24 hr. Urine samples were collected in four different bottles that correspond to 0–2, 2–6, 6–12, and 12–24 hr after drinking. Fecal samples from 0–24 hr after chicory juice drinking were collected.

### 2.4 Phenolic acids in chicory, serum, urine, and fecal samples

Phenolic acids in chicory and biological samples were extracted and determined by a high‐performance liquid chromatography assay with electrochemical detection (HPLC‐ECD) as previously described with minor modifications (Wang, Liu, Zheng, Fan, & Cao, 2011). The column was a Zorbax SB‐C18 column (250 mm × 4.6 mm, 5.0 µm). The mobile phase adopted was 50% methanol (A) and 2% aqueous acetic acid (B) (v/v) using a linear gradient elution of 5%‐20% A at 0–10 min, 20%‐40% A at 10–15 min, 40%‐60% A at 15–25 min, and 60%‐70% A at 25–35 min. Quantification of phenolic acids was carried out by an internal standard method using calibration curves. Aliquots of biological samples (serum, urine, and feces) from each participant were treated according to one of the three following procedures: no treatment (to detect free phenolic acids), β‐glucuronidase (to detect glucuronide phenolic acids), or sulfatase in the presence of d‐saccharic acid 1,4‐lactone (to detect sulfate phenolic acids) (Shelnutt, Cimino, Wiggins, Ronis, & Badger, 2002). For each analytical run, a standard curve was prepared in the appropriate matrix (methanol, blank serum, urine, or feces) and used to determine the content of phenolic acids in chicory or biological samples. The detection limit for phenolic acids (10‐fold baseline noise) under the conditions used in this study was 1 ng/ml in serum and 5 ng/ml in urine and feces, and values below this concentration were reported as zero. Recoveries of protocatechuic acid were 93.7% in serum, 89.5% in urine, and 83.9% in feces. The intra‐ and interday variations were below 8.05% in serum, urine, and feces. Mycophenolic acid was selected as the internal standard.

### 2.5 Catabolism of phenolic acids by human fecal suspensions

The in vitro fermentation of phenolic acids with human fecal suspensions was performed as we have previously described (Lin, Wang, Yang, Wang, & Ling, 2016). Autoclaved brain–heart infusion medium was supplemented with phylloquinone, heme, and l‐cystine and was placed in an anaerobic chamber for 48 hr prior to the fermentation to reduce oxygen. Fresh feces (2 g) collected from human adults were mixed with 10 ml of brain–heart infusion medium, and the resulting solution was filtered with autoclaved gauze to remove the dregs before use. Next, the fecal suspension (100 µl) and 1 µmol of each tested standard compound (gallic, caffeic, 5‐caffeoylquinic, caftaric, or chicoric acid) were added to 4 ml of brain–heart infusion medium. The mixture was fermented at 37°C under anaerobic conditions using the anaerobic chamber. Samples were taken after 0, 6, 12, and 24 hr of fermentation and acidified with 30 µl of 6 M HCl to inactivate the microbiota before being stored at − 80°C. All incubations were performed in triplicate. At the same time, the fecal suspension was incubated without phenolic acids as a negative control.

### 2.6 Pharmacokinetic analysis

The pharmacokinetic parameters of protocatechuic acid in the free, glucuronide, and sulfate conjugates were calculated using DAS 2.1 (BioGuider Co.), with a noncompartmental model: maximum serum concentrations (C_max_); time to achieve maximum serum concentrations (T_max_); the area under the concentration–time curve to 24 hr (AUC_0‐24_); and terminal elimination half‐life (T_1/2z_). The apparent bioavailability of protocatechuic acid together was quantified using AUC_0‐24_ divided by its ingested amount. Urinary and fecal recoveries were calculated using the total content of urinary and fecal protocatechuic acid divided by its ingested amount.

### 2.7 Statistical analysis

Data are presented as means ± *SD*. The significance of differences between the baseline (0 hr) and the indicated time points after chicory consumption was assessed by ANOVA for repeated measures and Dunnett's 2‐tailed *t* test, assuming the baseline values as the reference category. *p < .05* was considered significant.

## 3 RESULTS

### 3.1 Phenolic acids in chicory

**Figure 2:**
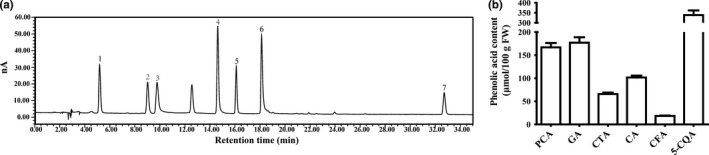
Characteristics of the chicory phenolic acids. (a) A representative profile of chicory phenolic acid separations obtained by HPLC‐ECD. Peaks identification: 1, gallic acid; 2, protocatechuic acid; 3, caftaric acid; 4, 5‐caffeoylquinic acid; 5, caffeic acid; 6, chicoric acid; and 7, mycophenolic acid. Mycophenolic acid was served as an internal standard. (b) Content of the phenolic acids in chicory. Values are the means ± *SD*, *n* = 6. GA, gallic acid; PCA, protocatechuic acid; CTA, caftaric acid; 5‐CQA, 5‐caffeoylquinic acid; CFA, caffeic acid; CA, chicoric acid; MPA, mycophenolic acid. FW, fresh weight

Phenolic acids consist of derivatives of hydroxybenzoic acids and hydroxycinnamic acids. Representative HPLC chromatograms of phenolic acid extracts from chicory are shown in Figure 2a. Hydroxybenzoic acids including protocatechuic (166.9 ± 8.7 µmol/100 g FW) and gallic acid (177.0 ± 10.8 µmol/100 g FW), and hydroxycinnamic acids including caffeic (18.3 ± 0.8 µmol/100 g FW), 5‐caffeoylquinic (338.3 ± 21.2 µmol/100 g FW), caftaric (65.8 ± 2.9 µmol/100 g FW), and chicoric acid (101.5 ± 3.8 µmol/100 g FW) were identified in the chicory (Figure 2b).

### 3.2 Participant characterization

All participants, which included eight men and eight women, were generally healthy with normal blood biochemistry and body mass index (Table S2).

### 3.3 Serum protocatechuic acid and its metabolites

**Figure 3:**
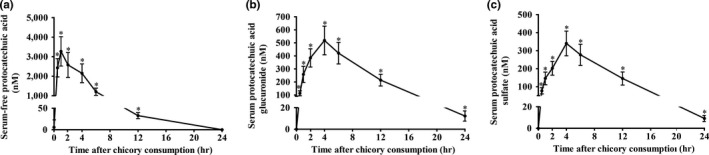
Serum concentrations of protocatechuic acid and metabolites over 24 hr in 16 healthy adults after consumption of 150 g of chicory. (a‐c) Serum concentration–time curves (0–24 hr) of protocatechuic acid in the free (a)‐, glucuronide (b)‐, and sulfate (c)‐conjugated forms. Values are the means ± *SD*, *n* = 16. *Different from baseline (0 hr), *p < .05*

Before chicory consumption, the serum concentrations of protocatechuic acid in the free‐conjugated and its glucuronide/sulfate‐conjugated forms were close to zero. The serum concentrations of total protocatechuic acid were rapidly increased at 0.5 hr after chicory juice consumption. The serum concentration–time curve of free protocatechuic acid showed for its compounds a C_max_ of 3,273 ± 729 nmol/L at 1 hr postconsumption (Figure 3a; Table 1). Both glucuronide‐ and sulfate‐conjugated protocatechuic acid peaked at 4 hr and then decreased to the basal level at 24 hr after chicory juice consumption (Figure 3b‐c; Table 1). The T_1/2z_ of serum‐free protocatechuic acid was 1.72 ± 0.35 hr, while that of serum glucuronide and sulfate conjugates was 6.12 ± 1.09 hr and 5.42 ± 1.12 hr (Table 1), respectively. The recovery of total protocatechuic acid from the blood circulation was approximately 23.79% of the ingested dose. Of note, glucuronide and sulfate conjugates accounted for about 21.11% and 13.68% of total recovered protocatechuic acid in the blood circulation (Table 2), respectively.

**Table 1:** Pharmacokinetic characteristics of protocatechuic acid and metabolites in serum over 24 hr in 16 healthy adults after consumption of 150 g of chicory

Compounds	C_max_ (nmol.L^−1^)	T_max_ (h)	T_1/2z_ (h)	AUC_0−24_ (µmol.h.L^−1^)
PCA‐F	3,273 ± 729	1	1.72 ± 0.35	16.90 ± 3.00
PCA‐G	519 ± 106	4	6.12 ± 1.09	5.54 ± 0.99
PCA‐S	340 ± 67	4	5.42 ± 1.12	3.60 ± 0.68

Note

Values are the means ± *SD*, *n* = 16.

Abbreviations: F, free; G, glucuronide; PCA, protocatechuic acid; S, sulfate. C_max_, maximum serum concentrations; T_max_, time to achieve maximum serum concentrations; T_1/2z_, terminal elimination half‐life; AUC_0‐24_, area under the concentration–time curve to 24 hr.

**Table 2:** Recovery of protocatechuic acid and metabolites in biological samples in 16 healthy adults after consumption of 150 g of chicory

Compounds	Blood circulation (% of the ingested dose)	Urine (% of the ingested dose)	Feces (% of the ingested dose)
PCA‐F	15.60% ± 4.21%	5.02% ± 3.24%	3.25% ± 1.36%
PCA‐G	4.96% ± 1.19%	5.65% ± 2.35%	7.77% ± 3.21%
PCA‐S	3.23% ± 0.81%	1.49% ± 0.85%	1.77% ± 0.90%
Total	23.79% ± 5.56%	12.17% ± 5.72%	12.79% ± 5.29%

Note

Values are the means ± *SD*, *n* = 16.

Abbreviations: F, free; G, glucuronide; PCA, protocatechuic acid; S, sulfate.

### 3.4 Urinary excretion of protocatechuic acid and metabolites

**Figure 4:**
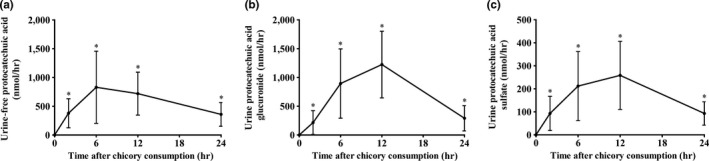
Urine concentrations of protocatechuic acid and metabolites over 24 hr in 16 healthy adults after consumption of 150 g of chicory. (a‐c) Urine excretion of protocatechuic acid in the free (a)‐, glucuronide (b)‐, and sulfate (c)‐conjugated forms. Values are the means ± *SD*, *n* = 16. *Different from baseline (0 hr), *p < .05*

Before chicory consumption, all urine samples did not contain detectable protocatechuic acid in the free‐ and glucuronide/sulfate‐conjugated forms. All samples up to 24 hr after chicory juice consumption contained free protocatechuic acid and its glucuronide/sulfate‐conjugated form (Figure 4). Urinary excretion of free protocatechuic acid and its glucuronide/sulfate conjugates reached the plateau at 6 hr and 12 hr after chicory juice consumption (Figure 4), respectively. The recovery of total protocatechuic acid in urine was approximately 12.17% of the ingested dose (Table 2).

### 3.5 Fecal excretion of protocatechuic acid and metabolites

**Figure 5:**
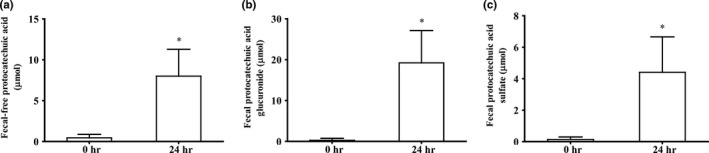
Fecal contents of protocatechuic acid and metabolites over 24 hr in 16 healthy adults after consumption of 150 g of chicory. (a‐c) Fecal content of protocatechuic acid in the free (a)‐, glucuronide (b)‐, and sulfate (c)‐conjugated forms. Values are the means ± *SD*, *n* = 16. *Different from baseline (0 hr), *p < .05*

At baseline, the concentrations of protocatechuic acid in the free‐ and glucuronide/sulfate‐conjugated form in fecal samples were close to zero. Chicory juice consumption increased the levels of free protocatechuic acid and its glucuronide/sulfate conjugates. The mean contents of free‐, glucuronide‐, and sulfate‐conjugated forms of protocatechuic acid in feces were 8.00 ± 3.17 µmol, 19.22 ± 7.65 µmol, and 4.41 ± 2.18 µmol (Figure 5), respectively. The recovery of total protocatechuic acid in feces was approximately 12.79% of the ingested dose (Table 2).

### 3.6 Pharmacokinetics of protocatechuic acid between men and women

When data for men and women were analyzed separately, some pharmacokinetic parameters differed (Table 3). The serum T_1/2z_ of sulfated conjugate of protocatechuic acid tended to be higher in women than in men (Table 3). Furthermore, the recovery of glucuronidated conjugate of protocatechuic acid in urine was higher in women than in men (Table 3). Additionally, the recovery of free form of protocatechuic acid in feces was higher in women than in men. In contrast, the serum C_max_, T_max,_ and AUC_0‐24_, as well as total recoveries of protocatechuic acid in the blood circulation, urine, and feces, did not differ between men and women (Table 3).

**Table 3:** Pharmacokinetic parameters of protocatechuic acid in serum and recovery of protocatechuic acid in biological samples between men and women after consumption of 150 g of chicory

Parameters	PCA‐F		PCA‐G		PCA‐S		PCA‐Total	
	Men	Women	Men	Women	Men	Women	Men	Women
n	8	8	8	8	8	8	8	8
C_max_ (nmol.L^−1^)	3,098 ± 568	3,448 ± 823	520 ± 90	517 ± 121	336 ± 57	343 ± 75	3,484 ± 545	3,868 ± 892
T_max_ (hour)	1	1	4	4	4	4	1	1
T_1/2z_ (hours)	1.63 ± 0.04	1.81 ± 0.48	6.26 ± 0.75	5.98 ± 1.33	4.87 ± 0.75	5.97 ± 1.16*	2.73 ± 0.17	2.80 ± 0.28
AUC_0−24_ (µ mol.h.L^−1^)	16.59 ± 1.63	17.21 ± 1.81	5.49 ± 0.79	5.60 ± 1.15	3.59 ± 0.42	3.62 ± 0.87	25.56 ± 1.54	25.35 ± 5.38
Blood circulation (% of the ingested dose)	17.18 ± 4.05	14.01 ± 3.74	5.46 ± 0.85	4.46 ± 1.26	3.58 ± 0.53	2.88 ± 0.89	26.22 ± 4.18	21.35 ± 5.69
Urine (% of the ingested dose)	5.66 ± 3.87	4.38 ± 2.28	4.52 ± 2.21	6.79 ± 1.89*	1.13 ± 0.62	1.85 ± 0.89	11.31 ± 6.40	13.02 ± 4.79
Feces (% of the ingested dose)	2.52 ± 0.87	3.97 ± 1.38*	6.82 ± 2.89	8.71 ± 3.23	1.74 ± 0.91	1.81 ± 0.88	11.08 ± 4.57	14.49 ± 5.41

Note

Values are the means ± *SD*, *n* = 8.

Abbreviations: F, free; G, glucuronide; PCA, protocatechuic acid; S, sulfate. C_max_, maximum serum concentrations; T_max_, time to achieve maximum serum concentrations; T_1/2z_, terminal elimination half‐life; AUC_0‐24_, area under the concentration–time curve to 24 hr.

* Different from men, *p < .05*.

### 3.7 Catabolism of phenolic acids by human fecal suspensions

Gallic, caffeic, 5‐caffeoylquinic, caftaric, or chicoric acid (1 µmol) was incubated in fresh human fecal suspensions with gut microbiota, and the content of protocatechuic acid was then quantified. The initial substrate dose was 1 µmol, from which 95.8 ± 3.3%, 86.0 ± 3.0%, 79.3 ± 5.7%, 91.5% ± 4.7%, and 72.8 ± 4.0% for gallic, caffeic, 5‐caffeoylquinic, caftaric, and chicoric acid, respectively, were recovered at the initial time point (0 hr) from the fecal suspensions (Table 4). While all tested phenolic acids were gradually degraded to varying degrees in 24 hr, protocatechuic acid could not be detected in fresh human fecal suspensions with gut microbiota (Table 4).

**Table 4:** Time courses of the formation of the metabolite protocatechuic acid in human fecal suspensions

Compound (µmol)	Incubation time (h)			
	0	6	12	24
*Gallic acid*	0.96 ± 0.03	0.75 ± 0.14*	0.60 ± 0.18*	0.27 ± 0.23*
Protocatechuic acid	ND	ND	ND	ND
*Caffeic acid*	0.86 ± 0.03	0.16 ± 0.11*	ND	ND
Protocatechuic acid	ND	ND	ND	ND
*5‐caffeoylquinic acid*	0.79 ± 0.06	ND	ND	ND
Protocatechuic acid	ND	ND	ND	ND
*Caftaric acid*	0.92 ± 0.05	ND	ND	ND
Protocatechuic acid	ND	ND	ND	ND
*Chicoric acid*	0.73 ± 0.04	ND	ND	ND
Protocatechuic acid	ND	ND	ND	ND

Note

Values are the means ± *SD*, *n* = 6; ND, not detected.

* *p < .05* compared with the *T*_0_ groups.

## 4 DISCUSSION

Several human studies have consistently shown that protocatechuic acid produced in the lower gastrointestinal tract by microbial catabolism of certain flavonoids (e.g., anthocyanins, procyanidins, and quercetin) is highly bioavailable (Czank et al., 2013; McKay et al., 2015; Urpi‐Sarda et al., 2009; Vitaglione et al., 2007). Because the upper and lower gastrointestinal tract has different potency in the absorption and metabolism of dietary factors, the pharmacokinetics of protocatechuic acid in humans following its oral consumption remains unknown. In the current study, we evaluated the absorption, metabolism, and excretion of protocatechuic acid in sixteen healthy adults over a 24‐hr period after a single oral consumption of 150 g of chicory. We obtained four major findings: 1) The peak serum concentrations of free protocatechuic acid reached 3,273 nmol/L at 1 hr after chicory consumption; 2) the absorption of protocatechuic acid into the blood circulation was about 23.79%, which mainly consisted of its free form rather than glucuronide/sulfate conjugates; 3) 12.17% and 12.79% of the ingested protocatechuic acid were excreted in urine and feces, respectively; and 4) the recovery of total protocatechuic acid in the blood circulation, urine, and feces consisted of its glucuronidated and sulfated forms by 34.79%, 60.15%, and 72.70%, respectively. Those novel findings thus allowed us to propose that protocatechuic acid from chicory is bioavailable and undergoes partial glucuronidation and sulfation in healthy human adults.

Because in vitro studies have shown that protocatechuic acid and its glucuronide/sulfate conjugates at the dosage of 1,000 nmol/L exert anti‐atherosclerosis‐related effects in macrophage‐derived foam cells and vascular endothelial cells (Amin et al., 2015; Liu et al., 2016; Wang, Xia, et al., 2012; Wang, Zhang, Wang, Liu, & Xia, 2012), the peak serum concentrations of protocatechuic acid after chicory consumption (3,273 nmol/L) are noteworthy. More importantly, both chicory and its constitute protocatechuic acid have been documented to inhibit atherosclerosis in ApoE^‐/‐^ mice through similar mechanisms by promoting endothelium‐dependent vasodilation (Liu et al., 2016). Therefore, evaluating whether regular consumption of chicory exerts health‐promoting effects in humans, especially the anti‐atherosclerotic effect, is worthy of further exploration.

Previous studies in humans and animal models have shown that gut microbiota metabolism of certain polyphenols is able to generate protocatechuic acid (Czank et al., 2013; Lin et al., 2016; McKay et al., 2015; Serra et al., 2012; Urpi‐Sarda et al., 2009; Vitaglione et al., 2007). In addition to protocatechuic acid, chicory contains abundant gallic, caffeic, 5‐caffeoylquinic, caftaric, or chicoric acid. To test whether human gut microbiota metabolism of their compounds contributes to the pool of protocatechuic acid in the subjects after chicory consumption, we incubated gallic, caffeic, 5‐caffeoylquinic, caftaric, or chicoric acid with fresh human feces of healthy adults that have not consumed fruits, vegetables, and plant‐based beverages/wine for 48 hr. We found that their compounds were intensively degraded; however, protocatechuic acid was not generated. Consistently with our findings, Gonthier et al., 2006 reported that human gut microbiota metabolism of caffeic, 5‐caffeoylquinic, and caftaric acid cannot generate protocatechuic acid. It should be pointed out that chicory also contains several flavonoids such as quercetin and apigenin (Ferioli, Manco, & D'Antuono, 2015). Our previous studies have demonstrated that quercetin and apigenin are intensively catabolized into protocatechuic acid by mouse gut microbiota (Lin et al., 2016). However, the contents of quercetin and apigenin in chicory are very limited, each of which is below 0.36 mg/100 g FW (Ferioli et al., 2015). Taken together, those findings allowed us to propose that protocatechuic acid other than phenolic acids (gallic, caffeic, 5‐caffeoylquinic acid, caftaric, or chicoric acid) and flavonoids (quercetin, apigenin) in chicory mainly contributes to the pool of protocatechuic acid in the subjects after chicory consumption.

Glucuronidation and sulfation are the most important phase II metabolic pathways for polyphenols (Manach, Scalbert, Morand, Remesy, & Jimenez, 2004). Because glucuronidation and sulfation significantly impact the physiological properties of polyphenols such as the solubility, intestinal absorption, tissue distribution, and excretion, the health‐promoting activities of polyphenols in vivo might be partially related to their glucuronide and sulfate conjugates. Supporting this possibility, several glucuronide and sulfate conjugates of polyphenols have been documented to exert biological activities (Polycarpou et al., 2013; Teles, Souza, & Souza, 2018; Williamson, Kay, & Crozier, 2018). Our current study showed that protocatechuic acid undergoes partial glucuronidation and sulfation evidenced by their presence in the blood circulation, urine, and feces in humans after chicory consumption. The recovery of total protocatechuic acid in the blood circulation, urine, and feces consisted of its glucuronide/sulfate by 34.79%, 60.15%, and 72.70%, respectively. Moreover, the peak serum concentrations of glucuronide and sulfate conjugates of protocatechuic acid reached 519 and 340 nmol/L, respectively. Both glucuronide and sulfate conjugates of protocatechuic acid at 100 nmol/L were recently documented to remarkably inhibit inflammation response in human umbilical vein endothelial cells stimulated with either oxidized LDL or a cluster of differentiation 40 ligand (Amin et al., 2015). To this end, it is logical to propose that in addition to free protocatechuic acid, glucuronidated and sulfated protocatechuic acid might elicit health‐promoting effects in human adults consuming 150 g of chicory.

Although the pharmacokinetics of polyphenols in humans has been well studied, little is known about their differences between men and women (Lu & Anderson, 1998). Our current data showed that three pharmacokinetic parameters including serum T_1/2z_ of sulfated conjugate, recoveries of urinary glucuronide conjugate, and fecal‐free form of protocatechuic acid were significantly different between men and women. These data highlighted that much work is needed to be accomplished in the issue of gender‐dependent pharmacokinetics of polyphenols in humans, which might help to explain the variable health‐promoting efficacy between men and women (Campesi, Marino, Cipolletti, Romani, & Franconi, 2018; Hughes et al., 2008).

Two limitations in this study should be born in mind. Firstly, although our findings from the in vitro fermentation assays showed that gallic, caffeic, 5‐caffeoylquinic, or chicoric acid could not be converted to protocatechuic acid by fresh human feces with gut microbiota, we did not exclude the possibility that the conversion of those phenolic acids to protocatechuic acid happens in humans owing to the fact that only a small proportion of gut microbiota can be cultivated in vitro (Gilbert et al., 2018). Secondly, 48.74% of the ingested protocatechuic acid was recovered in the blood circulation, urine, and feces; however, the remaining of the ingested protocatechuic acid was undetectable. In addition to glucuronidation and sulfation, methylation is known to be another common metabolic pathway for polyphenols in vivo (Manach et al., 2004). Indeed, studies conducted in rats have shown that protocatechuic acid can be methylated into vanillic acid (Cao et al., 2009). Therefore, it is possible that the undetectable protocatechuic acid in the systemic circulation, urine, and feces is present in its methylated form.

## 5 CONCLUSION

In conclusion, we have demonstrated for the first time that protocatechuic acid is bioavailable and undergoes partial glucuronidation and sulfation in healthy human adults following chicory consumption. Because protocatechuic acid possesses multiple biological effects in animal models, future clinical trials are worthy to confirm whether chicory recapitulates its effects in humans.

## CONFLICT OF INTEREST

None.

## AUTHOR CONTRIBUTIONS

D.W. conceived and designed the experiments; J.Z., H.X., L.H., H.W., and Q.L. performed experiments; J.Z, H.X., L.H., H.W., and Q.L. analyzed data; W.L. revised the manuscript; and D.W. and J.Z. wrote the paper. All authors read and commented the manuscript.

## ETHICAL APPROVAL

This study was approved by the Ethics Committee of the School of Public Health at Sun Yat‐sen University.
