# Supplementary material for: Protocatechuic acid from chicory is bioavailable and undergoes partial glucuronidation and sulfation in healthy humans
Source: Food Sci Nutr. 2019 Aug 14;7(9):3071–80. doi: 10.1002/fsn3.1168 (PMC6766590; doi:10.1002/fsn3.1168)
Supplement: Supplementary file 1 [file FSN3-7-3071-s001.docx]

**Supplemental Table 1.** Restricted foods rich in anthocyanins, phenolic acids or sesquiterpene lactones

| Category | Foods |
| --- | --- |
| Vegetables | Burdock root; Cabbage lettuce; Chicory; Chrysanthemum; Crown daisy; Chrysanthemum nankingense; Eggplant; Jerusalem artichoke; Lactuca sativa L; Lettuce; Olive; Onion; Purple cabbage; Purple yam; Red brassica campestris; Rhizoma dioscoreae; Spinach |
| Fruits | Apple; Blueberry; Blackcurrant; Blackberry; Cherry; Citrus; Grapefruit; Hawthorn; Kiwi; Lemon; Mulberry; Myrica rubra; Orange; Peach; Plum; Pomegranate; Red raspberry; Strawberry |
| Seeds | Beans |
| Cereals | Colored rice; Purple corn; Sorghum; Whole grain |
| Seasonings | Mint; Perilla nankinensis; Star anise |
| Beverages | Fruit juices (apple, blueberry, kiwi, lemon, grape, orange, pomegranate, strawberry); Beer; Tea; Wine |
| Others | Chocolate; Coffee; |

**Supplemental Table 2.** Characteristics of the participants^a^

| Subjects | Age  (y) | Weight  (kg) | Height  (cm) | BMI  (kg/m^2^) | SP  (mmHg) | DP  (mmHg) | AST  (U/L) | ALT  (U/L) |
| --- | --- | --- | --- | --- | --- | --- | --- | --- |
| Women | 41.75 ± 8.72 | 53.63 ± 3.67 | 157.63 ± 3.16 | 21.59 ± 1.69 | 113.75 ± 12.50 | 73.75 ± 7.07 | 20.95 ± 3.59 | 18.08 ± 1.50 |
| Men | 31.16 ± 3.88 | 65.00 ± 7.81 | 174.00 ± 4.36 | 21.38 ± 1.63 | 124.38 ± 5.54 | 71.25 ± 1.30 | 21.50 ± 2.63 | 20.57 ± 2.42 |

^a^Values are the means ± SD, n=8; BMI, body mass index; SP, systolic pressure; DP, diastolic pressure; AST, aspartate aminotransferase; ALT, alanine aminotransferase.
